# The Small Fibrinopeptide Bβ_15–42_ as Renoprotective Agent Preserving the Endothelial and Vascular Integrity in Early Ischemia Reperfusion Injury in the Mouse Kidney

**DOI:** 10.1371/journal.pone.0084432

**Published:** 2014-01-02

**Authors:** Anja Urbschat, Kai Zacharowski, Nicholas Obermüller, Katrin Rupprecht, Daniela Penzkofer, Carla Jennewein, Nguyen Tran, Bertram Scheller, Stefanie Dimmeler, Patrick Paulus

**Affiliations:** 1 Faculty of Medicine, Goethe-University Hospital, Frankfurt am Main, Germany; 2 Clinic of Anesthesiology, Intensive Care Medicine and Pain Therapy, Goethe-University Hospital, Frankfurt am Main, Germany; 3 Clinic of Internal Medicine III, Division of Nephrology, Goethe-University Hospital, Frankfurt am Main, Germany; 4 Institute of Cardiovascular Regeneration, Goethe-University, Frankfurt am Main, Germany; University of Kentucky, United States of America

## Abstract

Disruption of the renal endothelial integrity is pivotal for the development of a vascular leak, tissue edema and consequently acute kidney injury. Kidney ischemia amplifies endothelial activation and up-regulation of pro-inflammatory mechanisms. After restoring a sufficient blood flow, the kidney is damaged through complex pathomechanisms that are classically referred to as ischemia and reperfusion injury, where the disruption of the inter-endothelial connections seems to be a crucial step in this pathomechanism. Focusing on the molecular cell-cell interaction, the fibrinopeptide Bβ_15–42_ prevents vascular leakage by stabilizing these inter-endothelial junctions. The peptide associates with vascular endothelial-cadherin, thus preventing early kidney dysfunction by preserving blood perfusion efficacy, edema formation and thus organ dysfunction. We intended to demonstrate the early therapeutic benefit of intravenously administered Bβ_15–42_ in a mouse model of renal ischemia and reperfusion. After 30 minutes of ischemia, the fibrinopeptide Bβ_15–42_ was administered intravenously before reperfusion was commenced for 1 and 3 hours. We show that Bβ_15–42_ alleviates early functional and morphological kidney damage as soon as 1 h and 3 h after ischemia and reperfusion. Mice treated with Bβ_15–42_ displayed a significantly reduced loss of VE-cadherin, indicating a conserved endothelial barrier leading to less neutrophil infiltration which in turn resulted in significantly reduced structural renal damage. The significant reduction in tissue and serum neutrophil gelatinase-associated lipocalin levels reinforced our findings. Moreover, renal perfusion analysis by color duplex sonography revealed that Bβ_15–42_ treatment preserved resistive indices and even improved blood velocity. Our data demonstrate the efficacy of early therapeutic intervention using the fibrinopeptide Bβ_15–42_ in the treatment of acute kidney injury resulting from ischemia and reperfusion. In this context Bβ_15–42_ may act as a potent renoprotective agent by preserving the endothelial and vascular integrity.

## Introduction

Ischemia reperfusion (IR) injury is one of the leading causes for the clinical manifestation of acute kidney injury and its dramatic consequences. It is based upon a complex interplay between vascular, tubular and inflammatory factors [1]. However, severe sepsis, cardiac surgery, renal surgery and kidney transplantation are mostly responsible for temporary or prolonged ischemia. These pathologic states result in kidney misperfusion, and thus lead to acute kidney injury (AKI). The vulnerability of the kidney is highlighted by the fact that it is one of the first organs to fail in septic patients [2]–[4].

On a molecular anatomical basis, AKI is mainly caused by endothelial dysfunction of the renal vascular bed. Tight structural connections between individual endothelial cells are responsible for creating a united cell structure promoting vascular-tissue barrier. One of the most important endothelial anchor proteins is VE-cadherin. It is directly connected to the actin-based cytoskeleton and is one of the key molecules integrating and tightening endothelial cell junctions [5]–[7]. Upon pathologic stimuli, these tight connections are opened and VE-cadherin is degraded [8]. This breakdown of VE-cadherin-mediated endothelial barrier function leads to altered vascular permeability and remodeling [9]. Besides its reciprocal binding activity, VE-cadherin also possesses binding sites for other molecules, specifically distinct fibrin degradation products, which occur in large concentrations following impaired of the blood flow [10].

Bβ_15–42_ is a 28 amino acid cleavage product of fibrin. Following thrombin-induced fibrin formation it is released by fibrin E1 fragments through the action of plasmin [11]. This peptide proved able to prevent VE-cadherin disruption, and hence micro-vascular dysfunction, thereby exhibiting organo-protective characteristics [12], [13]. The fact that VE-cadherin shows affinity to the N-terminus of the fibrin Bβ-chain is especially noteworthy. Portions of the third and fourth extracellular domains of VE-cadherin constitute a receptor for the Bβ-chain of fibrinogen [14], [15]. Moreover, the peptide Bβ_15–42_ competes with other fibrin degradation peptides for binding to VE-cadherin, thereby preventing fibrinogen-induced trans-endothelial migration of leukocytes in IR injury [9], [11]. Given this important interaction with VE-cadherin, a therapeutic potential in preserving the vascular barrier in the disease state can be assumed [15], [16].

These vasculo-protective effects of Bβ_15–42_, as previously described by our research group, prompted us to test its therapeutic potential in the early phase of renal IR injury using the standard model of bilateral kidney IR in mice. In this setting, we evaluated whether Bβ_15–42_ is able to rapidly stabilize the endothelial barrier function when administered immediately prior to reperfusion. As a physiologically occurring fibrinopeptide, Bβ_15–42_ might represent a therapeutic option for the protection of kidneys during and after ischemic periods, a presumption that is substantiated by the results of the present experiments.

## Materials and Methods

### Animals

Male adult C57/BL6 mice (Janvier, St. Berthevin, France) weighing 25–30 g were housed in the central research facility of the Goethe-University Frankfurt. The mice were kept in approved plastic cages, had water and food ad libitum and were housed in rooms equipped with a 12 h light cycle. All procedures involving animals were approved by the Animal Care and Use Committee of the federal state of Hesse (Regierungspräsidium Darmstadt, Germany, file number: V54-19c20/15-F35/04). Surgical procedures and animal care were performed in accordance with the “Guide for the care and use of laboratory animals” (National Institutes of Health, volume 25, no. 28, revised 1996), EU Directive 86/609 EEC and Germany’s Protection of Animals Act.

### Interventional Groups

Before mice were subjected to a 30 min period of bilateral renal ischemia, randomization into four groups (for each n = 8) was performed: two sham-treated groups with i.v. application of saline at the beginning of reperfusion followed by 30 min (designated as: NaCl 1 h of IR) and 2.5 h (NaCl 3 h IR) of observation and two groups with i.v. application of the fibrin fragment Bβ_15–42_ at the beginning of reperfusion followed equally by a 30 min (Bβ 1 h of IR) and 2.5 h observation period (Bβ 3 h IR). Upon completion of the corresponding observation periods, animals were sacrificed, the complete kidneys carefully removed and divided longitudinally into halves. One half of which was placed in 4% paraformaldehyde overnight; the other, together with blood samples, was stored at −80°C pending further processing.

### Induction of Bilateral Kidney Ischemia and Reperfusion

Mice were deeply anaesthetized with an intraperitoneal injection of ketamine (100 mg/kg body weight) and xylazine (5 mg/kg body weight). Injection anesthesia was used since broad evidence is available regarding the protective effects of volatile anesthetics like Iso-, En- or Sevoflurane during ischemia and reperfusion. The organ-toxic effects of ether made it unsuitable for our investigation. During anesthesia (for surgery and for sonography), the constant body temperature of 37°C was monitored using a rectal probe and temperature was maintained using a heating pad. To ensure a uniform hemodynamic pattern within and between all groups, kidney perfusion was controlled using color duplex sonography. After bilateral dorsal flank incision and preparation of the kidney arteries, mice were subjected to 30 min of renal ischemia followed by 30 min or 2.5 h of reperfusion. Clamping was performed under microscopic control using nontraumatic microvascular clamps with a jaw pressure of 85 g (Micro-Serrefine 8 mm, Fine Surgical Instruments, Germany). To prevent arterio-venous clamping, the correct placement of the clamps was controlled microscopically. Correctly clamped kidneys turned pale, whereas arterio-venously clamped kidneys had a hemorrhagic, deep purple aspect and had to be excluded from the experiment. During the whole surgical procedure, mice were kept on a heating pad to maintain constant body temperature. Immediately prior to the removal of the clamp, Bβ_15–42_ (2.4 mg/kg body weight) or saline was administered as a 0.1 ml bolus intravenously retrobulbary. Clamps were then released and the recovery of renal blood flow was visually monitored. Subsequently, incisions were closed in layers and mice were allowed to recover in a heated surrounding. Animals were sacrificed after 1 h and 3 h of completed ischemia and reperfusion as specified in the protocol.

### Reagents and Dosage

Prof. Petzelbauer, Medical University of Vienna, kindly provided recombinant Bβ_15–42_. A stock solution was prepared by dissolving the peptide at a concentration of 72 mg/ml in phosphate-buffered saline. Intravenous administration of a 0.1 ml stock solution was then performed using 2.4 mg/kg body weight via retrobulbary injection. The dosage was determined based on previously published studies by our group on the topic of myocardial ischemia-reperfusion [13], [16].

### Color Duplex Sonography and Renal Blood Flow Measurements

Color duplex sonography was performed with a Vevo 2100 imaging system (VisualSonics Inc., Toronto, Canada) equipped with high frequency (22–55 MHz) linear transducer. For this purpose, anaesthetized animals were analyzed immediately before commencing surgery and after the corresponding time points. Each animal was controlled for heart rate using an ECG and for spontaneous breathing using a breathing detector attached to the Vevo system. Animals’ body temperature was controlled via rectal probe. Each study consisted of 10 s video sequence recording. Blood flow profiles were recorded from the arcuate arteries using PW-mode and synchronized to the electrocardiogram-signature. End-systolic and end-diastolic renal blood velocities were determined using the VisualSonics software. For evaluation we calculated the resistive index from at least 15 flow curves per file. Resistive index was then calculated using the formula: (VO_syst._−VO_diast._)/VO_syst._×100%.

### Histopathological Examination

Paraformaldehyde-fixed (4%) and paraffin-embedded kidneys were prepared as 5 µm thick slices. Hematoxylin-eosin (HE) staining was performed according to standard protocols. Histological examinations were conducted in a blinded fashion; for each slide, 10 fields at 400fold magnification of the cortex and outer medulla were photographed. Histomorphological examination was performed by analyzing the amount of cellular cores per field and by evaluating the core morphology as an expression of cellular damage. For this purpose, images were taken with the Leica DM5000B microscope and analyzed using an automatized Matlab script (The Mathworks, Natick, MA, USA). The software determines the values of each pixel in the RGB space thus allowing measurement of the relative area occupied by the number of nuclei per image (blue staining). Quantification was done by setting blue colored pixels in proportion to the total number of colored pixels. Basic principles of this method have been previously published [17].

### Immunohistochemical Staining, Immunofluorescence and TUNEL Assay

Immunohistochemistry on neutrophils was performed using the Vectastain® ABC kit (Vector Laboratories, Inc., USA). Immunofluorescence was performed for the following molecules: VE-cadherin and CD31. For specific staining, slides were incubated with the primary antibody overnight at 4°C (neutrophil staining: rat anti-mouse Ly-6B.2, #MCA771GA, Serotec, Germany; rat anti-mouse VE-cadherin, #138101, BioLegend, San Diego, USA; rat anti-mouse CD31, #SZ31, Dianova, Hamburg, Germany). For signal amplification and detection of the neutrophil staining, a biotinylated secondary antibody (rabbit anti-ratIgG, DAKO, Germany) was incubated for 1 h at room temperature prior to incubation for 1 h with streptavidin-HRP complex (AXXORA, Lörrach, Germany) and subsequent DAB incubation for 5 min. Counterstaining was carried out with hematoxylin for 10 min. For signal detection of VE-cadherin and CD31, the secondary antibody (donkey anti-rat IgGAlexa 488, Jackson Immuno Research, USA) was incubated for 1 h at room temperature.

Nuclear staining was performed using DAPI staining according to the manufacturer’s protocol. The TUNEL assay was performed according to the manufactor’s protocol (In situ cell death detection kit POD, #11684817910, Roche, Basel, Switzerland). Analysis of the slides was performed in a blinded fashion. For each slide, 10 randomly chosen fields at 400fold magnification were photographed from the cortex and outer medulla. Neutrophils were counted in a blinded fashion. Analysis for TUNEL and immunofluorescence were performed using an automatized Matlab script (The Mathworks, Natick, MA, USA) as described above. Quantification was done by setting blue colored pixels in proportion to brown colored pixels (TUNEL) and green colored pixels in proportion to the entire photographed area (VE-cadherin and CD31).

### Real-time RT-PCR

Total RNA was isolated from homogenized whole kidney samples using TRI Reagent (Sigma-Aldrich, USA) according to the manufacturer’s protocol. Then cDNA was synthesized using iScriptcDNA Synthesis kit (Bio-Rad laboratories, USA). Gene expression profiles of 18 s, P-Selectin, ICAM-1, and NGAL were assessed by quantitative real-time polymerase chain reaction using a StepOnePlus Realtime PCR device (Applied Biosystems, USA). In brief: after a denaturation phase of 10 s at 95°C, followed by an annealing phase at 60° for 10 s, a synthesis step at 72° for 25 s was performed. Specificity was detected by adding a melting curve procedure to the PCR. Primer sequences are listed in *Table 1*. Results were expressed as % of the control group (NaCl 1 h).

**Table 1:** Real-time PCR primers used for the quantification of mRNA expression levels.

Genes	Forward(5′–3′)	Reverse (5′–3′)
18 s	GTAACCCGTTGAACCCCATT	CCATCCAATCGGTAGTAGCG
P-Selectin	ACGGTACCATGTCCCCAAGCT	CCAGCGCTCGTGGAATCTCTC
ICAM-1	GAGCGGCGTCGAGCCTAGG	TCTCGTCCAGCCGAGGACCAT
NGAL	CCAGGGCTGGCCAGTTCACTC	TGGGTCTCTGCGCATCCCAGT

### Western Blot Analysis and ELISA

Proteins were isolated according to a standard protocol [17], [18]. 7.5% SDS-gels were loaded with 50 µg protein. Proteins were detected on Hybond C supermembrane (Amersham Pharmacia Biotech, UK) with Spectra brood range marker (Fermentas, Germany) as a standard. The blots were probed with antibodies against VE-Cadherin (Abcam, UK) and CXCR4 (Abcam, UK). Development was performed using a HRP-conjugated secondary antibody (Thermo Scientific, USA) and the ECL Western Blotting Substrate (Promega, Germany). Digitalization and evaluation of the blots were performed with a Kodak Imager (Carestream, Germany). For serum sampling, blood was withdrawn from the inferior vena cava at harvest. Samples were centrifuged at 10000xg for 10 min and serum supernatants were stored at −80°C for further processing. ELISA on mouse NGAL (R&D Systems Inc., Quantikinine® ELISA, USA) was carried out according to the manufacturer’s protocol. Readout was performed using a microplate reader (Bio-Tek Instruments, Germany).

### Statistical Analysis

Statistical analysis was performed with GraphPad Prism® 5.02 software (GraphPad Software, Inc., USA). Results are expressed as means ± standard error of the mean (SEM). Statistical significance was calculated using one-way ANOVA followed by Bonferroni’s multiple comparison test and Student’s t test. Statistical significance was set to p<0.05. Detection of the range of normal resistive index values was performed using a frequency distribution test, indicating the 25^th^ and 75^th^ percentiles as lower and upper limits of the normal values’ distribution.

## Results

### Fibrin-derived Peptide Bβ_15–42_ Attenuates Cellular Damage and Apoptosis in the Early Phase of Ischemia and Reperfusion

In order to morphologically quantify tissue damage and structural renal changes, 10 randomly taken pictures from each slide (n = 8/group) were analyzed counting the total number of nuclei and cell core areas using H&E staining. For detection of apoptosis rates TUNEL-staining was performed and equally 10 randomly taken pictures from each slide (n = 8/group) were analyzed focusing on the cortex and outer medulla.

**Figure 1:**
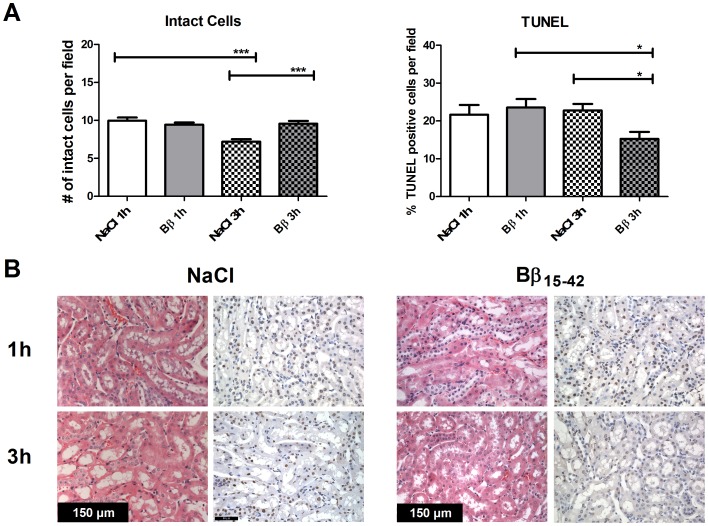
Bβ_15–42_ attenuates cellular damage and apoptosis after early renal ischemia and reperfusion. (**A**) Quantification of intact cells remaining within the groups (left graph) and quantification of the apoptosis rates (right graphs). (**B**) Representative micrographs of H&E (left micrographs) and TUNEL (right micrographs) stained kidney sections of Bβ_15–42_ and untreated mice at 1 h and 3 h after IR.10 fields within the cortex and outer medulla in each group (n = 8) have been evaluated at 400fold magnification. Quantification was performed using an automatized Matlab script measuring proportional colored pixels in a defined relative area. For H&E staining (left graph) quantification was done by setting blue to the total number of colored pixels. For TUNEL staining (right graph) brown was set into relationship to blue colored pixels. Error bars represent means ± SEM.*P<0.05, ***P<0.001; one-way ANOVA followed by Bonferroni’s multiple comparison test.

Overall, H&E staining revealed, that the number of intact nuclei remained nearly stable in Bβ_15–42_ treated mice after 1 h and 3 h of IR (9.44 resp. ±9.57 cellular cores/field; ns) reflecting better conserved micro-anatomic appearance of their kidneys. In untreated mice the number of intact nuclei declined over time (9.95±0.43 vs. 7.21±0.31 cellular cores/field; P<0.001). Bβ_15–42_ treated mice displayed increased numbers of intact nuclei, and thus intact cells, than sham-treated mice after 3 h of IR (9.57±0.36 vs. 7.21 cellular cores/field ±0.31; P<0.001), a similar tendency was observed after 1 h IR of IR (9.44±0.28 vs. 9.95±0.43 cellular cores/field; ns). (Fig. 1A,B left graph & left micrographs).

In TUNEL staining, animals receiving Bβ_15–42_ showed lower apoptosis rates than sham-treated mice after 3 h IR (15.29±1.79 vs. 22.78±1.70% TUNEL positive cells; P<0.05). Notably, the apoptosis rate decreased in Bβ_15–42_ treated mice from 1 h to 3 h of IR (23.54±2.22 vs. 15.29±1.79% TUNEL positive cells; P<0.05), while levels in sham-treated mice remained relatively unchanged in elevation during the observed time span (21.65±2.57 vs. 22.78±1.70% TUNEL positive cells; ns). No significant differences in apoptosis rates were seen at 1 h of IR between untreated and treated animals (21.65±2.57 vs. 23.54±2.22% TUNEL positive cells; ns). (Fig. 1A,B right graph & right micrographs).

### Preservation of VE-Cadherin in Mice Treated with Bβ_15–42_

To analyze to which extent Bβ_15–42_ inhibits VE-Cadherin disruption and subsequent degradation we performed VE-Cadherin western blot analyses and immunohistochemical staining for the detection of VE-Cadherin as well as CD31 for an additional visualization of the endothelium.

**Figure 2:**
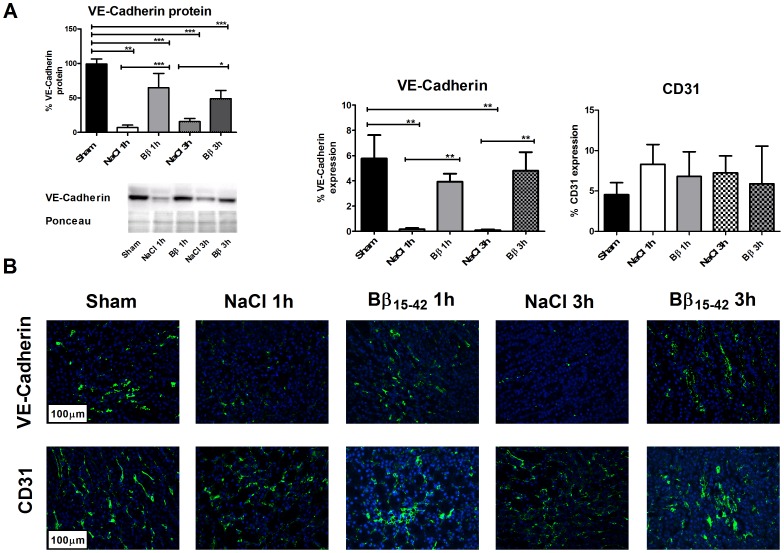
Significant preservation of VE-Cadherin in mice kidneys treated with Bβ_15–42_. (**A**) Western blot immunoassays of renal homogenates on VE-Cadherin (left graph) and immunofluorescence double-staining of the area occupied by VE-Cadherin and CD13 (right graphs), respectively.10 fields within the cortex and outer medulla in each group (n = 8) have been evaluated at 400fold magnification. Data are expressed as the ratios of VE-Cadherin (green) positive vs. VE-Cadherin negative areas per field and CD13 (green) positive vs. CD13 negative areas per field. (**B**) Representative micrographs of selective immunofluorescence staining on VE-Cadherin (upper micrographs) and CD13 (lower micrographs) of all groups (sham, NaCl 1 h, Bβ 1 h, NaCl 3 h, and Bβ 3 h). Error bars represent means ± SEM. *P<0.05, **P<0.01, ***P<0.001. Values are expressed in % expression. One-way ANOVA followed by Bonferroni’s multiple comparison test.

In the corresponding immunoblots, VE-Cadherin is highly conserved in animals receiving Bβ_15–42_ after 1 h (64.75±12.10 vs. 7.16±2.02% VE-Cadherin expression; P<0.001) and 3 h of IR (48.89±6.88 vs. 15.82±2.52% VE-Cadherin expression; P = 0.05) when compared to the corresponding controls. (Fig. 2A, left graph).

Similarly, in immunohistochemical staining, we could confirm this general loss of VE-Cadherin in the ischemic kidney of NaCl treated mice after 1 h and 3 h of IR in comparison to sham-operated mice (0.16±0.09 and 0.09±0.06 vs. 5.77±1.85% VE-Cadherin expression; P = 0.01 and P<0.01). In contrast, Bβ_15–42_ treated mice displayed no significant alteration of VE-Cadherin levels at 1 h and 3 h of IR (3.93±0.63 and 4.80±1.46 vs. 5.77±1.85% VE-Cadherin expression; ns and ns). Comparing saline treated and Bβ_15–42_ treated mice a reduced loss of VE-Cadherin in Bβ_15–42_ treated mice could be observed at both time points (1 h: 0.16±0.09 vs. 3.93±0.63% VE-Cadherin expression; P<0.01) (3 h: 0.09±0.06 vs. 4.80±1.46% VE-Cadherin expression; P<0.01). (Fig. 2A,B right graph & upper micrographs).

CD31 displayed no significant alteration at both time points in sham-treated and ischemic kidneys (ns). (Fig. 2A,B right graph & lower micrographs).

### Pro-inflammatory CXCR4 Protein Expression as well as Renal Neutrophil Infiltration is Reduced in Bβ_15–42_ Treated Mice

**Figure 3:**
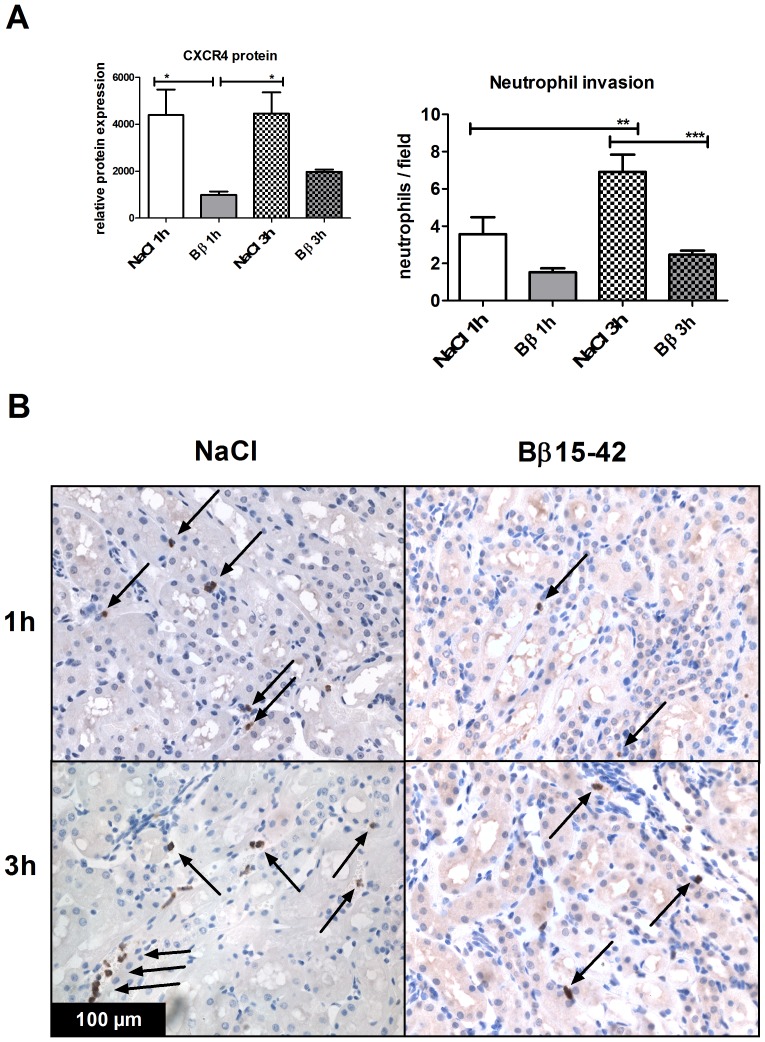
Significantly reduced neutrophil infiltration and pro-inflammatory CXCR4 protein expression in Bβ_15–42_ treated mice kidneys in early renal ischemia and reperfusion. (**A**) Western blot immunoassays of renal homogenates on CXCR4 (left graph) and neutrophil counting (right graph). (**B**) specific neutrophil staining (brown cells with arrows). 10 fields within the cortex and outer medulla in each group (n = 8) have been evaluated at 400fold magnification. Data are expressed as number of neutrophils/field. Error bars represent means ± SEM. *P<0.05, **P<0.01, ***P<0.001; one-way ANOVA followed by Bonferroni’s multiple comparison test.

The neutrophil regulator CXCR4 is significantly reduced in animals that received Bβ_15–42_ after 1 h (993.80±139.60 vs. 4400±1088 relative protein expression; P<0.05) and 3 h of IR (1976±96.55 vs. 4464±904.20 relative protein expression; P<0.05) when compared to the corresponding controls. (Fig. 3A).

Neutrophil infiltration within the cortex and outer medulla of the injured renal tissue was assessed by immunohistochemistry. We observed a tendency to fewer neutrophils in Bβ_15–42_ treated mice compared to controls after 1 h (1.53±0.21 vs. 3.57±0.91 neutrophils; ns) with a significant difference at 3 h of IR (2.47±0.22 vs. 6.91±0.92 neutrophils; P = 0.001). Over the time, the number of neutrophils increased significantly in untreated mice from 1 h to 3 h of IR (3.57±0.91 vs. 6.91±0.92 neutrophils; P = 0.01), while the increase in Bβ_15–42_ treated mice was less pronounced (1.53±0.21 vs. 2.47±0.21 neutrophils; ns). (Fig. 3A,B).

### Bβ_15–42_ Treatment Reduces P-Selectin and ICAM-1 Gene Expression

We examined the gene expression of P-Selectin and ICAM-1, as selective recruitment of circulating leukocytes into the sites of injury is mediated by primary (selectin) and activation-dependent (integrin) adhesion molecules.

**Figure 4:**
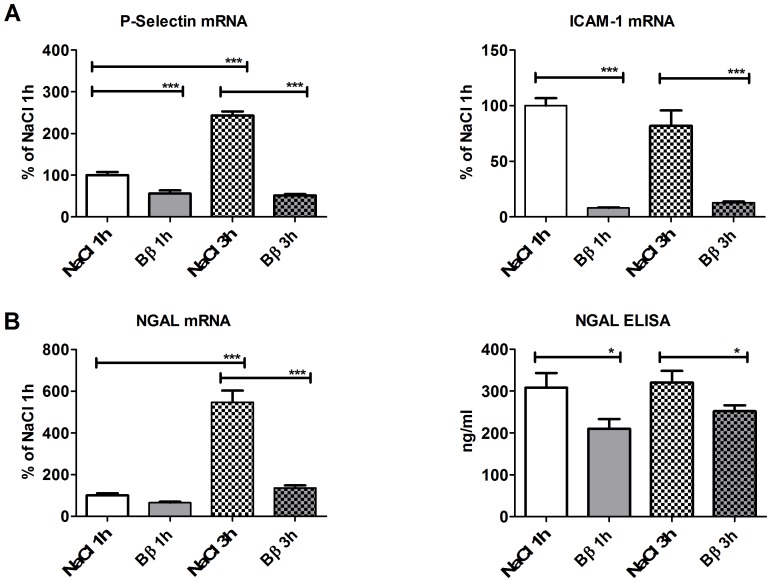
AKI-marker NGAL serum levels and tissue gene expression of P-Selectin and ICAM-1 as well as NGAL were significantly reduced after IR in mice treated with Bβ_15–42_ compared to untreated mice. (**A**) Tissue gene expression of adhesion molecules P-Selectin (left graph) and ICAM-1 (right graph). (**B**) Tissue gene expression of the AKI marker NGAL (left graph). Circulating protein levels of serum NGAL (right graph). Values are expressed in % of NaCl for mRNA analyses and in ng/ml for ELISA measurements. All experiments were performed in duplicates. Error bars represent means ± SEM. *P<0.05, ***P<0.001. One-way ANOVA followed by Bonferroni’s multiple comparison test and unpaired t-test (ELISA).

P-Selectin mRNA expression was significantly reduced in Bβ_15–42_ treated mice compared to untreated subjects after both 1 h (56.08±7.34 vs. 100.00±7.48% of NaCl 1 h; P<0.001) and 3 h of IR (51.37±3.51 vs. 243.00±9.94% of NaCl 1 h; P<0.001). During the time-span of 1 h to 3 h of IR, P-Selectin mRNA significantly increased in untreated mice (100.00±7.48 vs. 243.00±9.94% of NaCl 1 h; P<0.001), whereas mice treated with Bβ_15–42_ mRNA levels remained nearly stable during the same period (56.08±7.34 vs. 51.73±3.51% of NaCl 1 h; ns). (Fig. 4A).

ICAM-1 gene expression remained consistently very low in mice treated with Bβ_15–42_ after 1 h (8.08±0.49 vs. 100.00±6.76% of NaCl 1 h; P<0.001) and 3 h of IR (12.81±1.05 vs. 81.83±13.88% of NaCl 1 h; P<0.001) compared to respective levels in saline-treated mice. (Fig. 4A).

### In Bβ_15–42_ Treated Mice the Acute Kidney Injury Marker NGAL is Significantly Reduced Regarding Gene Expression and Serum Levels

Neutrophil gelatinase-associated lipocalin (NGAL) a biomarker of early ischemic acute kidney injury was assessed to investigate the effect of Bβ_15–42_ on renal function. As the gold standard, serum creatinine, displays poor specificity and sensitivity with regard to recognition of the early period of acute kidney injury [19]–[21] NGAL gene expression in tissue homogenates as well as serum NGAL was used for kidney function assessment [22], [23].

First, we found that NGAL mRNA expression was reduced in Bβ_15–42_ treated mice compared to untreated mice after 1 h IR (65.95±4.46 vs. 100.00±10.82% of NaCl 1 h; ns); a finding that was much more striking at 3 h IR (136.20±12.22 vs. 547.30±55.60% of NaCl 1 h; P<0.001). Over time, from 1 h to 3 h of IR, NGAL expression rose significantly in untreated mice (100.00±10.82 vs. 547.30±55.60% of NaCl 1 h; P<001), whilst the increase in treated mice was less pronounced (65.95±4.46 vs. 136.20±12.22% of NaCl 1 h; ns). (Fig. 4B).

Moreover, animals treated with Bβ_15–42_ displayed a significantly lower increase of serum NGAL levels compared to sham-treated animals after 1 h (210.09±23.50 vs. 308.55±34.83 ng/ml; P<0.05) and 3 h of IR (252.15±13.89 vs. 320.88±27.62 ng/ml; P<0.05). Over time, serum NGAL levels remained high in untreated mice at 1 h and 3 h (308.55±34.83 vs. 320.88±27.62 ng/ml; ns), whereas only a modest rise in serum NGAL levels could be observed in Bβ_15–42_ treated mice (210.09±23.50 vs. 252.15±13.89 ng/ml; ns). (Fig. 4B).

### Bβ_15–42_mice Display a Better Preserved Vascular Function After IR

**Figure 5:**
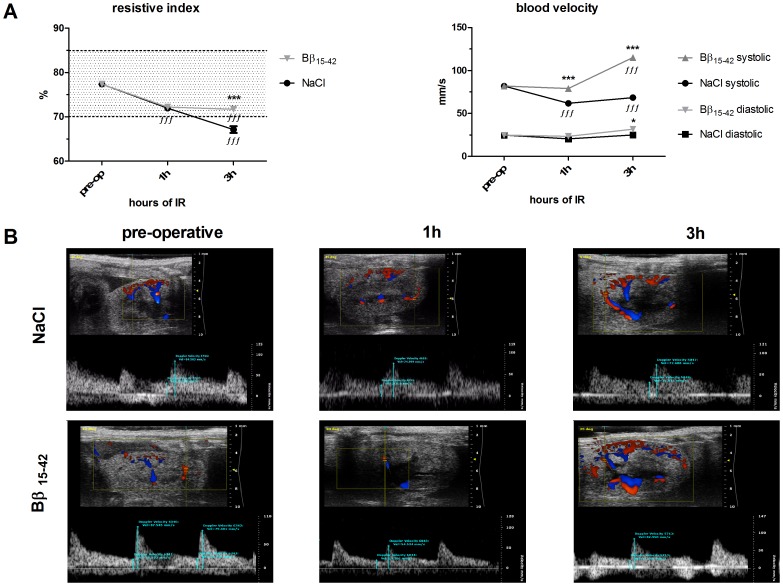
Better preserved vascular function in Bβ_15–42_ treated mice after IR. (**A**) Kidney perfusion was evaluated by determining the resistive index in %. (left graph) and blood velocity in mm/s (right graph). Dotted lines indicate upper and lower normal limits (left graph). Systolic (upper lines) and diastolic (lower lines) values are plotted (right graph). Measurements were performed on 10 s video sequences by evaluating at least 15flow signatures per animal. (**B**) Representative pictures of duplex sonography measurements performed pre-operatively, 1 h and 3 h after completed IR in NaCl (upper micrographs) and Bβ_15–42_ (lower micrographs) treated mice.*Significantly different from mice treated with saline; ∫ significantly different from pre-operative measurements. Time points and error bars represent means ± SEM. ∫∫∫ and ***P<0.001; one-way ANOVA followed by Bonferroni’s multiple comparison test.

To determine whether application of Bβ_15–42_ impacts renal function, we directly measured functional vascular kidney parameters using color duplex sonography. The resistive index (RI) determines the resistance of the intra-renal arterial system. Usually either an increase in vascular resistance or hypotension leads to increased RI values. First we observed an immediate and significant drop in the RI of mice treated with NaCl already at 1 h of IR (71.98±0.59%; P<0.001), which continued to drop at 3 h of IR (67.14±0.77%; P<0.001) in comparison to pre-operative measurements (77.39±0.43%). In Bβ_15–42_ treated mice the RI declined comparable to NaCl treated mice at 1 h of IR (72.21±0.44% vs. pre-operative measurements 77.39±0.43%; P<0.001). After 3 h of IR though, in Bβ_15–42_ treated mice the RI remained nearly stable but still below pre-operative measurements (71.73±0.52% vs. 77.39±0.43%; ns). Measured values for resistive index ranged from 60.16% to 94.93% in preoperative animals. The 25% percentile was 70.10% indicating the lower normal value and the 75% percentile was 84.92% indicating the upper normal value for mouse resistive index variability, assuming a Gaussian distribution. (Fig. 5A,B left graph & lower pictures).

Secondly, we determined the blood velocity within the arcuate arteries as direct parameter for renal perfusion. Animals treated with NaCl displayed significantly lower systolic blood velocities after 1 h and 3 h of IR compared to the pre-operative values (61.87±1.26 (1 h) vs. 81.91±1.88 mm/s (pre-operative); P<0.001) (68.42±1.24 (3 h) vs. 81.91±1.88 mm/s (pre-operative); P<0.001). In contrast, the diastolic blood velocity remained relatively unchanged over time compared to pre-operative measurements (20.38±0.47 (1 h) vs. 24.77±1.07 mm/s (pre-operative); ns) (25.00±0.74 (3 h) vs. 24.77±1.07 mm/s (pre-operative); ns). Interestingly, mice treated with Bβ_15–42_ generally displayed a higher blood velocity than mice treated with NaCl. Systolic blood velocity remained nearly unchanged after 1 h of IR compared to pre-operative measurements (79.07±1.64 (1 h) vs. 81.91±1.88 mm/s (pre-operative); ns) but increased significantly at 3 h of IR (115.2±2.99 (3 h) vs. 81.91±1.88 mm/s (pre-operative); P<0.001). In Bβ_15–42_ treated mice the diastolic blood velocity remained relatively unchanged at 1 h of IR (23.38±0.52 (1 h) vs. 24.77±1.07 mm/s (pre-operative); ns) and raised moderately at 3 h of IR (31.67±0.76 (3 h) vs. 24.77±1.07 mm/s (pre-operative); ns). These findings, demonstrating a higher perfusion in animals treated with Bβ_15–42_ at reperfusion, suggest that Bβ_15–42_ might directly be involved in the regulation of post ischemic kidney perfusion. (Fig. 5A,B right micrograph and lower pictures).

## Discussion

Endothelial integrity is essential for the maintenance of the vascular barrier [24]. Under hemodynamic disturbances, as observed in temporary ischemia and reperfusion, this integrity is impaired and may even lead to tissue injury in the kidney [25]. Severe IR injury can cause tubular endothelial cell necrosis, principally of cells within the cortex and outer medulla, resulting in acute tubular necrosis (ATN), clinically referred to as acute kidney injury (AKI) [26]. Specifically, before attached leukocytes may transmigrate through the endothelial layer, the EC-EC junction, formed by VE-cadherin, has to be loosened [27]. If bound to VE-cadherin, fibrin fragments mask the binding sites that can interact with leukocytes, thus preventing endothelial attachment and consecutive transmigration [28]. In this context, the fibrinopeptide Bβ_15–42_ inhibits endothelial leukocyte adhesion and thus exerts an anti-inflammatory effect [11], [13], [16].

In our histological examination, we quantified tissue damage, structural renal changes and apoptosis. Animals that received Bβ_15–42_ displayed significantly lower apoptosis rates and a higher number of intact cells than untreated mice after 3 h of ischemia and reperfusion. Histomorphological analyses revealed that apoptosis mostly occurs in proximal tubular cells, whereas the number of endothelial cells remains mostly unchanged (Fig. 2). Thus, we assume that these profound structural changes occurring in IR injury of the kidney are effectively alleviated by administration of Bβ_15–42_.

Several studies have shown that early post-IR neutrophil invasion modulation might represent an option for renal protection by preventing disruption of the above-described micro-anatomic entity [29]–[31]. Here, neutrophil invasion is described as one of the most potent triggers of tissue injury. Neutrophil adhesion, which precedes invasion, involves the CXC-motif-chemokine receptor *(*CXCR)-4 receptor [32] and requires an interaction with surface molecules that are upregulated on activated endothelium. Selectins bind to other carbohydrate-containing counter-receptors to induce the rolling of neutrophils on endothelium, the initial step in attachment, and therefore represent key mediators of neutrophil trafficking and hemostasis [33] contributing to the overall extent of AKI [34]. ICAM-1, an adhesion receptor expressed on endothelial cells finally determines neutrophil and leukocyte-endothelial cell adhesion and is known to be expressed by the kidney endothelium following renal ischemia [29]. These data are in line with our findings, since we equally observed a significant post-ischemic up-regulation of CXCR4 protein, P-Selectin and ICAM-1 mRNA expression (Fig. 3,4). Upon administration of Bβ_15–42_ at the time of reperfusion, CXCR4 increased significantly slower after 1 h and the physiologic increase of P-Selectin and ICAM-1 mRNA expression was significantly reduced at 1 h and 3 h after ischemia reperfusion. Consistent with this decreased endothelial activation, we detected a significantly reduced neutrophil infiltration in Bβ_15–42_ treated mice after 3 h of ischemia and reperfusion, which we attribute to the “sealed” endothelial barrier (Fig. 3).

This hypothesis is strengthened by our findings concerning the inter-endothelial adhesion molecule VE-Cadherin. Here we observed a significant degradation of this important junctional molecule in NaCl treated animals over time, which in turn indicates a consecutive loss of the barrier function, allowing neutrophils to pass. Yet, in Bβ_15–42_ treated mice VE-cadherin remained intact conserving its function as a junctional molecule (Fig. 2).

We therefore can provide evidence that first IR-induced kidney damage occurs already at a very early stage and secondly that early treatment with Bβ_15–42_ is able to stabilize endothelial barrier in the tubulo-vascular compartment of the cortex and outer medulla.

The resistive index has clinically been described to reflect intra-renal resistance [35]. Values below normal may indicate vasoplegia, values above vasoconstriction. Thus changes in blood flow or perfusion are related to endothelial dysfunction or damage. Assessing renal perfusion using color duplex sonography, we were able to show that upon Bβ_15–42_ treatment, perfusion remained efficient (as determined by resistive index), whereas in NaCl treated animals, perfusion dropped significantly over time. The reason for this pathologic state might be a vasoplegia in NaCl treated animals starting to occur in the first hours of reperfusion as demonstrated by lower systolic blood velocities (Fig. 5). We thus conclude that functionally Bβ_15–42_ prevents from endothelial dysfunction. As hypothesized, Bβ_15–42_ stabilizes VE-Cadherin and thus correlates with better renal perfusion, indicating a conserved endothelial function. This, in turn, leads to the interruption of an incipient vicious circle that would consecutively induce the over-expression of adhesion molecules such as P-Selectin and ICAM-1, resulting in increased neutrophil infiltration. Whether the regulation of P-Selectin or ICAM-1 is directly or indirectly influenced by Bβ_15–42_, remains unclear at this point.

Additionally, we explored whether the expression of NGAL as novel early diagnostic and prognostic biomarker of AKI [19]–[21], [23], which is detectable in serum already within hours after IR [22], [36], is altered in our study. Initially mice treated with Bβ_15–42_ displayed a significantly lower increase in NGAL tissue gene expression (Fig. 4). These findings were additionally supported through the demonstration of significantly lower NGAL serum levels in these mice at just 1 h as well as 3 h after ischemia reperfusion. This finding reflects the better-preserved renal function upon treatment with Bβ_15–42_.

Our findings are congruent with the recently published data from Sörensen and colleagues indicating that Bβ_15–42_ attenuates the effect of ischemia reperfusion injury in renal transplantation [37]. Furthermore, the role and abundance of α, β and γ chains of fibrinogen was recently investigated in the setting of IR injury by Krishnamoorthy et al. [38]. The latter found that mRNA expression of fibrinogen β-chains increases in the rat and mouse kidney following IR injury. Whereas it was located in the renal interstitium in control mice, fibrinogen β-chains appeared in the peri-tubular epithelia cell in injury [38]. This is an important finding since Bβ_15–42_ corresponds to the VE-Cadherin binding sequence of the fibrin β-chain. This research group, however, focused on renal tissue repair and at the stages investigated (24 h, 48 h post-ischemia) no alterations in the expression of adhesion molecules, leukocyte infiltration nor apoptosis rate were detectable. In the present study, we were able to demonstrate that endothelial activation, disruption of inter-endothelial junctions together with trafficking of neutrophils into the site of injury, occurs very early, already during the first hours of reperfusion injury.

We therefore conclude that these time points represent pivotal moments for the initiation of tubular injury. Accordingly, already at this early stage exogenous administration of Bβ_15–42_ reduces IR-induced endothelial activation and degradation of the junction molecule VE-Cadherin, and hence diminishes inflammatory cell influx, subsequently leading to a better preserved integrity of the endothelium, less apoptosis and tissue damage and hence better preserved vascular perfusion in the very early phase of IR injury.

Taken together, our data indicate that IR injury related kidney damage initiates at a very early stage. We were able to demonstrate that endothelial dysfunction in the tubular system already manifests itself at just 1 h after reperfusion onset and can be significantly alleviated by early intervention at this stage. In this experimental setting, Bβ_15–42_ exerts an immediate protective effect when administered directly prior to reperfusion. Furthermore, we believe that this very early onset of interventions and consecutive reduction of acute kidney injury will be beneficial not only in terms of the acute alterations but may also have a concomitant positive impact with regard to the long-term effects of renal damage.
